# Reinforcement Strategies for Load-Bearing Calcium Phosphate Biocements

**DOI:** 10.3390/ma8052700

**Published:** 2015-05-20

**Authors:** Martha Geffers, Jürgen Groll, Uwe Gbureck

**Affiliations:** Department for Functional Materials in Medicine and Dentistry, University Hospital Würzburg, Pleicherwall 2, D-97070 Würzburg, Germany; E-Mails: martha.geffers@fmz.uni-wuerzburg.de (M.G.); juergen.groll@fmz.uni-wuerzburg.de (J.G.)

**Keywords:** calcium phosphate cements, porosity, fiber reinforcement, dual setting, mechanical properties

## Abstract

Calcium phosphate biocements based on calcium phosphate chemistry are well-established biomaterials for the repair of non-load bearing bone defects due to the brittle nature and low flexural strength of such cements. This article features reinforcement strategies of biocements based on various intrinsic or extrinsic material modifications to improve their strength and toughness. Altering particle size distribution in conjunction with using liquefiers reduces the amount of cement liquid necessary for cement paste preparation. This in turn decreases cement porosity and increases the mechanical performance, but does not change the brittle nature of the cements. The use of fibers may lead to a reinforcement of the matrix with a toughness increase of up to two orders of magnitude, but restricts at the same time cement injection for minimal invasive application techniques. A novel promising approach is the concept of dual-setting cements, in which a second hydrogel phase is simultaneously formed during setting, leading to more ductile cement–hydrogel composites with largely unaffected application properties.

## 1. Introduction

Self-setting cements based on calcium phosphate chemistry combine the advantages of the high biocompatibility of calcium phosphates with the free mouldability of cements and the mechanical stability of ceramic implants [1,2]. Such calcium phosphate cements (CPC) are usually based on freshly prepared mixtures of crystalline or amorphous calcium orthophosphate, calcium hydroxide or calcium carbonate powders with an aqueous solution, which undergo setting in a continuous dissolution–precipitation reaction. Although various mixtures of calcium and phosphate sources can serve as raw materials, there are in principle only two cement types as products of the setting reaction: At neutral or basic pH the calcium phosphate cement sets to nanocrystalline hydroxyapatite (HA, with a variable stoichiometric composition between Ca_9_(PO_4_)_5_HPO_4_OH–Ca_10_(PO_4_)_6_(OH)_2_), while at low pH < 4.2, orthophosphate ions are protonated and the secondary phosphates brushite (CaHPO_4_·2H_2_O, DCPD) and monetite (CaHPO_4_, DCPA) are the least soluble calcium phosphates [3,4] and hence precipitated during setting of acidic cement pastes until an end pH of close to 5 [1,2,5,6,7]. Detailed reviews about CPCs reflecting their synthesis, setting reaction, rheological properties or biological performance can be found in literature [2,8,9]. CPC are resorbed *in vivo* and replaced by new bone tissue [10,11], whereas the speed of degradation depends on the final composition of the cement matrix. Hydroxyapatite forming cements degrade only slowly within years since the surrounding extracellular fluid ([Ca^2+^] ~ 2.5 mmoL/L, [HPO_4_^2−^] ~ 1 mmoL/L [12]) is supersaturated regarding HA (solubility of hydroxyapatite ~ 0.2–0.3 mg/L) [2]. HA forming cements degrade solely by osteoclastic bone remodeling, which is limited to surface degradation since cells cannot penetrate the microporous cement structure. Osteoclastic cells resorb the cement by providing a local acidic environment increasing the solubility of the mineral [13,14,15,16]. In contrast, cements forming brushite or monetite have a higher solubility (calculated solubility in water for monetite: 41–48 mg/L, brushite: 85–88 mg/L [17]) and many studies have demonstrated the bone remodelling capacity of such cements in various animal models within a time period of 8–52 weeks [18,19,20,21]. A passive resorption of such cements by simple chemical dissolution is a topic of contention in the literature, whereas some authors postulate that the extracellular liquid is in equilibrium with brushite [22], while others have calculated a thermodynamic instability of brushite in simulated body fluid [23]. The latter is supported by the fact that brushite forming cements are indeed dissolved *in vivo* even in the absence of osteoclastic cells (e.g., after intramuscular implantation) [24]. Worth noting is that for brushite forming cements a phase transformation into lower soluble minerals like octacalcium phosphate, hydroxyapatite or whitlockite can occur *in vivo* by a dissolution–reprecipitation reaction, which slows down biodegradation [25,26].

**Figure 1:**
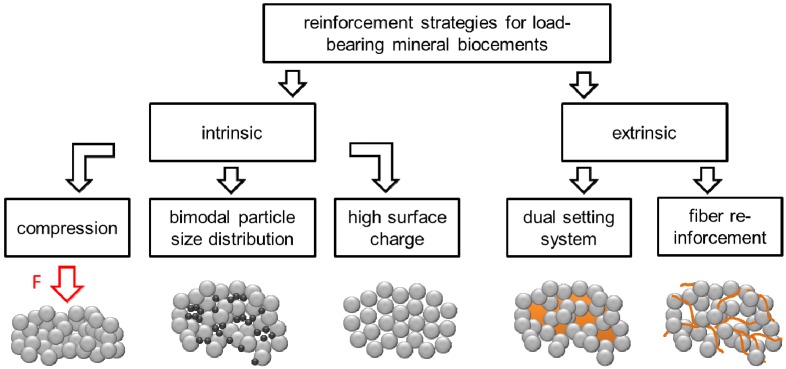
Strategies to reinforce mineral biocement for load-bearing applications.

Calcium phosphate bone cements have been shown to provide compressive strength of up to 80 MPa measured under application near conditions without a precompaction of the cement paste leading to lower porosity/higher strength, since this is not applicable under *in vivo* conditions [27]. Set CPC can be considered as porous ceramic materials with an inherent brittleness and comparatively low flexural strength compared to natural hard tissues such as bone or teeth. A comprehensive characterization of the elastic and failure properties for both hydroxyapatite and brushite forming CPC by Charrière *et al.* [28] indicated brushite cements to be suitable as bone fillers, while hydroxyapatite cements were attributed to having the potential to be a structural biomaterial. The low fracture toughness restricts the use of CPC to non-load-bearing defects [29]. Typical applications are the treatment of maxillofacial defects or deformities [1] or the repair of craniofacial defects [30]. An extension of the application of calcium phosphate cements to load-bearing defects, e.g., in vertebroplasty or kyphoplasty [31,32,33], would require less brittle cements with an increased fracture toughness. This is of high interest since the application of commonly used polymeric cements have strong drawbacks near the spinal cord due to their strong exothermic setting reaction and cytotoxic monomer release [34,35,36]. Common approaches to reduce brittleness of CPC and to improve their mechanical performance for load-bearing applications cover the modification of the cement liquid with polymeric additives such as collagen [37,38,39,40], the addition of fibres to the cement matrix [41,42] or the use of dual-setting cements in which a dissolved monomer is simultaneously cross-linked during cement setting [43,44,45] (Figure 1). This article aims to feature the most significant reinforcement strategies for calcium phosphate cements based on either intrinsic (porosity) or extrinsic (fiber addition, dual setting cement) material modifications.

## 2. Porosity Reduction for Strength Improvement of CPC

Calcium phosphate biocements set by a dissolution–precipitation reaction, during which the cement raw material continuously dissolves to form a supersaturated solution with regard to the setting product. The latter is precipitated from the aqueous cement phase and forms an entangled cementitious crystal matrix. The mechanical strength of a cement matrix is a direct result of this crystal entanglement and several factors determine the final strength of the matrix, such as degree of conversion, setting product or porosity. The latter is likely the most important factor and it is known from literature that porosity reduction in cements from 50% to 31% by compression can increase compressive strength by nearly an order of magnitude [46]. Porosity in biocements predominately originates from the presence of unreacted cement liquid after setting located in the voids between the entangled crystal matrix. Since any excess of water used for paste mixing, which is not consumed during the setting reaction creates porosity, the main influencing parameter on the total cement porosity is the powder to liquid ratio (PLR) used for cement processing. Pore sizes in CPC typically have a diameter range spanning from a few nanometers to several micrometers [47,48] and are occupying about 22–55 vol% of cements without further paste manipulation (e.g., compaction, porogen addition) [49,50]. Generally, pores in hydroxyapatite cements are smaller than in brushite cements (due to smaller crystal size of HA), whereas the total porosity is mostly smaller for brushite cement. The latter is a result of an increased water consumption during brushite cement setting.

Porosity considerably lowers the strength and stiffness (Young’s modulus) of the cements matrix with an inverse exponential relationship between cement porosity and compressive strength:

*CS* = *CS*_0_exp^~2K*P*^ (1)

where *CS* is the compressive strength at a given porosity; *CS*_0_ is the maximum theoretical strength of the material; K is a constant; and *P* is porosity [46]. Porosity is usually measured by helium pycnometry [51], mercury intrusion porosimetry (MIP) [52] or it is calculated based on the phase composition of the set cements and their densities [53]. Due to the disadvantages of these methods (destructive, long analysis times, toxicity of mercury, misleading results due to amorphous phases), Unosson *et al.* [54] have investigated a method which is based on the assumption that the evaporated water from a dried cement sample equals to the volume of pores within the cement. Since the accuracy of this method depends on a quantitative drying of samples without affecting the phase composition, the authors evaluated several drying conditions (vacuum, elevated temperature) for cement samples and compared the results with porosity determined by the above mentioned methods. Since the measured porosity was found to vary between the different methods, the authors recommended using more than one method to determine cement porosity, whereas the water evaporation method (24 h in vacuum) proved to be fast, easy and precise in estimating the porosity of CPCs.

**Figure 2:**
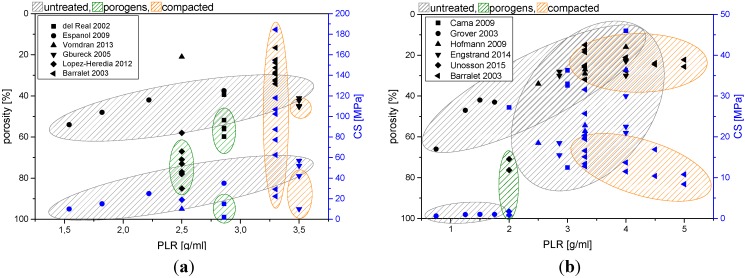
Correlation between powder to liquid ratio and porosity/compressive strength for (**a**) hydroxyapatite and (**b**) brushite cement from different studies. Cements were either set without compacting manipulation (untreated), processed by pre-compaction or porogens were added to create artificial macroporosity. Data were obtained from: (**a**) del Real 2002 [58], Espanol 2009 [47], Vorndran 2013 [59], Gbureck 2005 [27], Lopez-Heredia 2012 [60], Barralet 2003 [61], (**b**) Cama 2009 [62], Grover 2003 [51], Hofmann 2009 [50], Engstrand 2014 [49], Unosson 2015 [63], Barralet 2003 [64].

Porosity reduction by decreasing the amount of cement liquid used for mixing is a key parameter to increase the intrinsic strength of any biocement matrix. This, however, is limited, since every cement powder requires a formulation specific minimum amount of water (“plastic limit”) for surface wetting of all cement particles and for filling the space between the particles [55]. A correlation between the powder to liquid ratio used for forming a cement paste and the resulting porosity/compressive strength is displayed in Figure 2 for both HA and brushite forming cements. An effective method to reduce cement porosity is based on both creating a bimodal size distribution of cement raw materials and the creation of a high surface charge (zeta-potential) of the particles. A bimodal size distribution is thought to fill space in cement pastes normally occupied by water. The possibility to reduce porosity has been demonstrated for both hydroxyapatite [27] and brushite [49,50] forming biocements. In addition, a high surface charge (zeta-potential) will help to disperse agglomerates of fine sized particles by reducing attractive interparticulate forces. The zeta-potential can be influenced by using multiple charged ions as additives to the cement liquid, e.g., tatrates or citrates [56], which adsorb at the particle surface and increase the zeta-potential to values of ~−40 to −50 mV. Applying these two principles to a matrix of α-tricalcium phosphate (monomodal size distribution with d_50_ ~9.8 µm) by using 13–33 wt% fine sized CaHPO_4_ filler (d_50_ ~ 1.16 μm) and 0.5 M trisodium citrate solution increase the plastic limit of the cements from 3.5 to 5.0 g/mL. At the same time, porosity was decreased from 37% to 25% and a strength improvement from 50 to 79 MPa could be found [27]. Another study by Engstrand *et al.* [49] investigated the effect of β-TCP filler particles on the mechanical properties of a brushite forming cement (β-TCP-MCPM system). The results showed that the addition of low amounts of a filler (up to 10%) in combination with 0.8 M citric acid solution can effectively increase the powder to liquid ratio and hence decrease porosity from ~30% to ~23%. This strongly affects compressive strength of the cements with an increase from ~23 MPa (no filler and citric acid) to ~42 MPa. Space in cement pastes may also be filled by using hard agglomerates similar to civil engineering Portland cements as shown by Gu *et al.* [57]. In this study, the dispersion of 20% high-strength β-tricalcium phosphate granules with a size of 200–450 μm in the cement showed an increase of the compressive strength by 70%, while maintaining the rheological properties (injectability through 2.2 mm needle by applying a 5 kg weight on the syringe plunger) of the cement paste.

Caution must be exercised when comparing the obtained strength values from different studies, since many parameters during cement sample preparation and testing can affect the results. Unlike polymeric polymethylmethacrylate (PMMA) based bone cements [65], testing of calcium phosphate bone cement is not regulated, and our own experiences show that strength of set cement can vary by several times depending on the sample preparation and testing conditions. Generally, strength of dried samples is superior to that of (application near) wet specimen, mainly because water acts as a lubricant between the entangled crystals of the precipitated matrix. In addition, sample preparation may cause changes of cement porosity, e.g., by precompacting the paste in a mold. This ejects liquid from the paste (through the narrow gap between mold and plunger) leading to a lower porosity and hence a higher strength compared to uncompacted samples [61,66,67].

## 3. Fiber Reinforcement of CPC

Similar to reinforcement approaches of sintered hydroxyapatite ceramics [68], the addition of fibers to CPC is one of the most successful reinforcement technique [41,69]. The mechanical behavior of such fiber reinforced calcium phosphate cements (FRCPC) is a result of the complex interaction between all of the composite constituents. Contributions to the macroscopic behavior come from strength and stiffness of both fiber and cementitious matrix, matrix toughness, mechanical interaction between fibers and matrix as well as supplementary effects of polymeric additives or aggregates [69].

Fiber reinforcement studies have been performed with many different types of fibres (degradable *vs.* non-degradable, see Table 1 showing a strong increase of the mechanical strength depending on several parameters such as (1) matrix composition and strength, (2) fibre volume fraction, orientation, aspect ratio and tensile modulus as well as (3) the interface properties between matrix and fibres [69]. In addition to an increase of the bending strength from approx. 10–15 MPa for pure CPC to a maximum strength of 45 MPa (polyglactin fibers)—60 MPa (carbon fibers), especially the work of fracture for fiber reinforced cement composites usually increases by at least one order of magnitude (Table 1).

**Figure 3:**
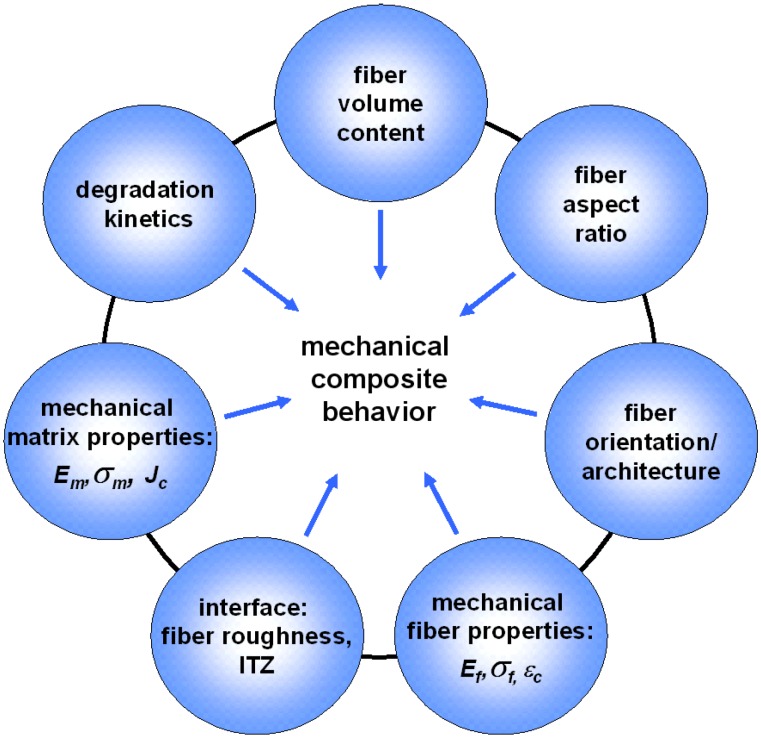
Interaction of material parameters which influence the time dependent mechanical behavior of the FRCPC composite. Reprinted with permission from [69].

As illustrated in Figure 3, there is not only a complex interaction of factors, but in clinical application the properties of the fiber–cement composites are also time dependent since both the cement matrix and the fibers may degrade during tissue regeneration.

Generally, the load-bearing capacity of fibers increases with their Young’s modulus, whereas the maximum tensile stress within the fiber is determined by the fiber’s modulus and the matrix strain [70]: 

(2) $$\sigma_{f}^{\max}=E_{f}\varepsilon_{m}$$

When the composite is loaded, differences between Young’s moduli of fiber and matrix lead to additional strain near the interface, mainly in the softer material [71]. The diameter of the fibers directly influences the total interface area between fibers and matrix for a given fiber volume fraction and affects both homogeneity and processability of the fiber–cement mixtures. Most biomedical composites are reinforced by discontinuous fibers. Their length and diameter are of great relevance, since substantial load has to be transferred from the matrix to the fiber via the interface for a reinforcing effect. Load is predominantly transferred by shear stresses at the lateral surface of the fibers rather than via the end faces of the fibers. Reinforcement effects are only observed, if the fiber length exceeds a critical value *l*_c_, which can be calculated based on the assumption that the fiber is loaded up to the fracture strength.

(3) $$l_{c}=\frac{d\sigma_{f,B}}{2\tau_{i}}$$

where σ_fB_ and τ_i_ denote fracture strength of the fiber and shear stress at the interface and d is the diameter of the fiber. Optimum fiber volume content has been addressed by many researchers. Civil engineering concretes typically are reinforced with <5 vol% of steel, glass, natural or synthetic polymer fibers [72]. In many studies on medical FRCPC, the fiber content is one order of magnitude higher than in fiber reinforced cements for civil engineering. This is attributed to a frequently observed trend in FRCPC research [73,74,75] that strength and ductility of the composites increased with fiber content. Moderate load transfer due to non-optimized interface strength and low modulus of the fibers require such high fiber volume fraction. Furthermore, fiber costs are not such a limiting factor, at least in the research stage.

**Table 1:** Examples for the reinforcement of calcium phosphate cements with either degradable or non-degradable fibres. (3 p.b.: Three point bending, 4 p. b.: Four point bending. ^#^ UD: Unidirectional fibers. TTCP: Tetracalcium phosphate. HA_w_: Hydroxyapatite whiskers) [69].

Composition Fiber/Additive/Matrix	Fiber Volume Fraction	Strength [MPa]	Work of Fracture [kJ/m²]	Test Method	Ref.
DEGRADABLE FIBRES					
HA matrix (TTCP + DCPA (+ Na_2_HPO_4_ − solution))	-	10–15	0.032–0.05	3 p. b.	[76,77]
Polyglactin 910/-/HA (TTCP + DCPA)	25 vol%	17.5–25	2.6–3.6	3 p. b.	[76]
Polyglactin 910/-/HA (TTCP + DCPA)	Mesh multilayer	8.5–24.5	0.75–3.1	3 p. b.	[78]
Polyglactin 910/chitosan lactate/HA (TTCP + DCPA)	45 vol%	41	11	3 p. b.	[74]
Polyglactin 910/chitosan lactate/HA (TTCP + DCPA)	Mesh multilayer	43	9.8	3 p. b.	[79]
Polyglactin 910/(poly(caprolactone))/brushite (β-TCP + H_3_PO_4_)	24 vol% random short 6–25 long fibers UD ^#^	7.5–20	n.a.	4 p. b.	[80]
NON-DEGRADABLE FIBRES					
Carbon/-/HA (TTCP + DCPA)	2–10 vol%	32–60	3.5–6.5	3 p. b.	[72]
CNT/-/HA (α-TCP + HA)	0.2–1.0 wt%	8.2–10.5	n.a.	3 p. b.	[81]
Aramid/-/macroporous HA (TTCP + DCPA + Na_2_HPO_4_)	6 vol%	7.5–13.5	0.8–6.5	3 p. b.	[75]
HA_w_/-/HA (TTCP + DCPA)	10–40 vol%	5.4–7.4	57–102	4 p. b.	[82]

Biodegradable polylactic-co-glycolic acid (PLGA) is one of the most frequently used reinforcement fiber materials for CPC. For a high fiber volume, considerable increase in bending strength has been reported, e.g., from 2.7 MPa (unreinforced CPC) to 17.7 MPa for CPC with 45 vol% polyglactin fibers [74]. This strengthening effect can be further enhanced to 40.5 MPa by incorporation of chitosan lactate into the matrix. The synergistic strengthening of the CPC by chitosan and fibers together is stronger than from either suture fibers or chitosan alone [74], which was explained by both a much stronger cement matrix after chitosan incorporation supporting the suture fibers to better resist cracking as well as an improved suture-matrix bonding [72,74]. Generally, strength increases with length to diameter aspect ratio of fibers, whereas occurrence of fiber aggregation leading to inhomogenities in fiber distribution represents the practical upper limit for the aspect ratio. Xu and co-workers [72] systematically varied the length of carbon fibers in HA cement and found a continuous increase of strength between 3 and 75 mm fiber length (aspect ratio of 1000 and 9000), which was followed by a strength decrease for 200 mm long fibers (aspect ratio 25,000). While the use of such long fibers strongly alters the workability of cement pastes and impedes a minimal invasive application by injection, cement pastes filled with short fibers have been demonstrated to maintain their injection properties up to a fiber length of 1 mm and a fiber volume of 7.5% [83].

Cements may also be modified by using fiber meshes instead of single fibers, especially in cases where biomechanical stresses will primarily be oriented linearly or biaxially to the cement implant. Meshes provide a strength enhancement (in linear or biaxial direction) beyond that of randomly directed fibers and have the advantage that even thin bony structures (e.g., malar, orbital bones) or extensive cranial deficiencies can be reconstructed [30,78,84]. Von Gonten [30] could demonstrate that such a polyglactin mesh–CPC composites have a similar work of fracture to PMMA cements up to seven days’ immersion in a buffered electrolyte, which was considered to have potential for structural repair of bone defects.

Most of the studies about FRCPC deal with both non-degradable fibers and with a poorly soluble hydroxyapatite cement matrix (see Table 1 and references [41,69]). This will initially result in long term stable cement composites with only minor changes of mechanical properties. However, even the slow matrix degradation by osteoclastic cells will dissect fibers in a longer time frame, which will be encapsulated in newly formed tissue with the possibility of foreign body reactions. Especially, approaches using technical fiber types (e.g., carbon fibers) or even carbon nanotubes are questionable regarding this point due to their low biocompatibility. The use of degradable fibers in FRCPC may solve this problem and the *in vivo* behavior of such FRCPC has been proven in various studies and is part of a recent review article by Krüger *et al.* [69]. However, at the same time, the use of degradable fibers will result in a time dependent loss of the reinforcement effect due to dissolution of the fibers in an aqueous environment. This effect of fiber degradation on the composite strength was simulated for polyglactin/PLGA fiber material by immersion of reinforced hydroxyapatite cement in a simulated physiological solution [73,74]. These studies confirmed a strength decrease of the reinforced composite after 4–6 weeks’ immersion [73], which could be compensated by a simultaneous chitosan infiltration of the cement matrix [74]. As a solution to the above mentioned problems of either a loss of mechanical properties during fiber degradation or the release of non-degraded fibers, the different degradation kinetics of fibers and cement matrix need to be adjusted. An approach is the use of more degradable cements based on the formation of dicalcium phosphate dehydrate (brushite) in conjunction with PLGA fibers [82]. Other promising works are dealing with degradable magnesium phosphate cements, which are reinforced with magnesium metal wires [85]. Especially, the latter provides strong reinforcement effects with a maximum bending strength of the composites of 139 MPa.

## 4. Dual Setting Cements

While the addition of non-reactive polymers (e.g., collagen, chitosan, hyaluronic acid, cellulose derivates) [37,38,39,86,87] is commonly used for improving cement cohesion or biological performance, it is only of small benefit for the mechanical cement performance. A reduction of the brittleness of CPC and an increase of strength can be achieved by using polymeric compounds which can be cross-linked by binding calcium ions due to a high density of either carboxylic acid or organic phosphate moieties in the polymer chain, e.g., polyacrylic acid [88,89,90,91,92], polymethyl vinyl ether maleic acid [89,93], poly[bis(carboxylatophenoxy)phosphazene] [94] or poly(vinyl phosphonate) [95]. Such polymer modified cements set both by the aforementioned dissolution–precipitation mechanism as well as by deprotonating the organic acid following the formation of intra- or inter-chained bonding Ca^2+^–Acid chelates [94] with a highly reactive cement component (mostly tetracalcium phosphate) from the cement powder. Processing of such polymer–cement composites is either possible by reacting an aqueous solution at ambient conditions with the cement powder or by reacting dry cement/polymer mixtures at elevated temperature/pressure in a solid state reaction.

**Figure 4:**
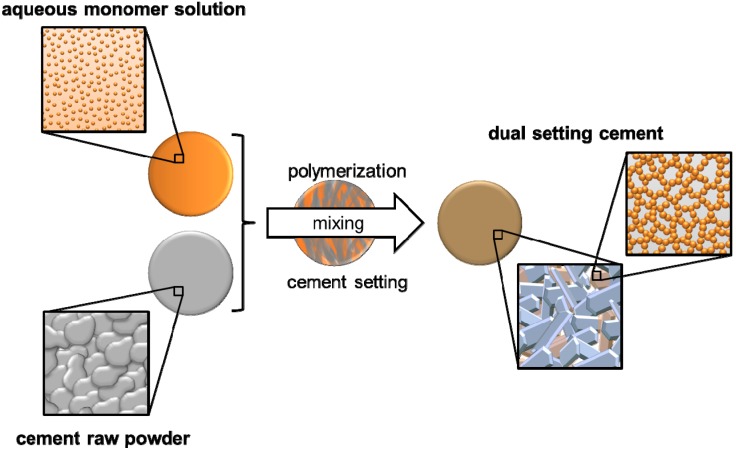
Hardening mechanism of dual-setting cements with the formation of interconnected matrices of hydrogel and precipitated cement crystals.

An alternative approach is the use of reactive monomer systems, which are dissolved in the cement liquid and simultaneously react during cement setting by a gelation–polymerisation process. This forms within several minutes a hydrogel matrix with embedded cement particles, which are subsequently converted into the setting product by a continuous dissolution–precipitation reaction. The result is finally an interconnecting hydrogel matrix within the porous cement structure as shown in Figure 4. The advantages of this strategy are the possibility of a high polymer loading of the cement (and hence a large strength and toughness increase) as well as practically unchanged rheological properties of the fresh cement paste. Both are related to the fact that the dissolved monomers are commonly small, water miscible liquids with low viscosity such that even high monomer concentrations are not strongly altering the initial cement viscosity.

An early study regarding this concept was using mixtures of triethyleneglycol-dimethacrylate (TEGDMA), Bisphenol-A-dimethacrylate (Bis-GMA), hydroxyethylmethacrylate (HEMA) and 10% water as cement liquid. After adding this liquid to an equimolar mixture of TTCP and DCPA, polymerization was initiated by benzoylperoxide (coating on cement particles) and di-(N,N)-2-hydroxyethyl-p-toluidine (added to the cement liquid) [96]. Although this study revealed high diametral tensile strengths of up to 26 MPa for such composites, no hydroxyapatite formation of the cement was observed even after 30 d storage in water. This was attributed to the low water content of the cement liquid as well as to an adsorption of the hydrogel on TTCP/DCPA cement particles. This problem was overcome by Dos Santos *et al.* [43,44,97], who modified the cement liquid of an α-tricalcium phosphate cement by the addition of 5%–20% acrylamide and 1% ammonium polyacrylate. While the latter was used to increase initial cement viscosity and to reduce cement wash out in an aqueous environment, the acrylamide was chemically polymerised during cement setting by the use of 0.25% of N,N,N',N'-tetramethylethylenediamide (TEMED) and 0.01% ammonium persulfate. This modification doubled the compressive strength of the set cement from 25–50 MPa while the tensile strength was increased from 9 MPa to <21 MPa. At the same time, the high water content of the cement liquid enabled setting of α-TCP cement particles to calcium deficient hydroxyapatite within seven days. A follow up study by the same authors extended the approach to a fiber reinforced–double setting cement matrix [43] by using 1–4 wt% of 4–10 mm long carbon, nylon and polypropylene fibers. The addition of the fibers was found to reduce the compressive strength of the cement, which was attributed to an increase of porosity. However, this was compensated by strong increase of the cement toughness and tensile strength, which increased from 17–28 MPa.

A major concern about this matrix is the toxicity of non-reacted acrylamide monomer. To overcome this problem, Christel *el al.* [98] investigated the modification of alpha-tricalcium phosphate cement (α-TCP) with 30%–70% of less-toxic 2-hydroxyethylmethacrylate (HEMA), which also resulted in mechanically stable polymer-ceramic composites with interpenetrating organic and inorganic networks. Four-point bending strength was found to increase from 9 MPa to more than 14 MPa when using 50% HEMA, and the bending modulus decreased from 18 GPa to approx. 4 GPa. In addition, cement composites with ≥50% HEMA showed strongly reduced brittle fracture behaviour with an increase of the work of fracture by more than an order of magnitude. While bending of pure ceramic samples was possible only to a maximum of 0.07 mm, samples with 50% or more HEMA monomer had a higher flexibility and bending was possible for 0.4–1.5 mm until fracture. At the same time, the authors could prove that important cement characteristics such as compressive strength or injectability were not significantly altered by using HEMA modification. Another study by Wang *et al.* [45] used methacrylate modified dextran as monomer in a cement matrix of tetracalcium phosphate/dicalcium phosphate anhydrous in a weight ratio of 10:1—1:3 (CPC: Meth.-dextran). The results showed an increase of the compressive strength from 24–83 MPa for a polymer content of 16.7%, as well as improvement of the fracture energy by nearly two orders of magnitude from 0.084–8.35 kJ·m^−2^.

Apart from using organic monomers to form a second network in cements, it is also possible to apply the concept of dual setting cements to pure inorganic materials. Silica addition to CPC is a common approach to modify bioactivity, cement paste cohesion and mechanical cement properties [99,100]. However, most studies either used non-reactive silica fillers in cements [101,102,103] or they added non-reactive calcium phosphate particles to an *in situ* forming silica matrix prepared by sol-gel processing [104,105,106]. In contrast, Geffers *et al.* [48] modified a brushite forming cement paste with a second inorganic silica based precursor, which was obtained by pre-hydrolysing tetraethyl orthosilicate (TEOS) under acidic conditions. The addition of the cement powder (mixture of β-tricalcium phosphate and monocalcium phosphate) provoked an increase of the pH of the silica precursor such that cement setting by a dissolution–precipitation process, and the condensation reaction of the hydrolysed TEOS occurred simultaneously. This resulted in an interpenetrating phase composite material in which the macro pores of the cement (pore sizes in μm range) were infiltrated by the micro porous silica gel (pore sizes in nm range), leading to a higher density and a compressive strength approximately 5–10 times higher than the CPC reference.

## 5. Conclusions and Outlook

This article features reinforcement strategies of biocements to improve their strength and toughness for an application at load-bearing defect sides. While porosity reduction is based on the optimization of an intrinsic cement property leading to higher strength, the addition of fibers or the creation of dual setting cement matrixes are extrinsic approaches not only improving strength but also toughness of the matrix. Surprisingly, most studies devoted to the mechanical properties of calcium phosphate biocements only deal with one of the presented strategies. Here, the simultaneous application of the different methods will definitely bring further improvements such that those optimized cements can likely be applied for load bearing defects. Desired mechanical properties would be likely similar to those of polymeric PMMA cements (bending strength ≥50 MPa, bending modulus ≥1800 MPa) compressive strength ≥70 MPa according to ISO 5833:2002 [107]), whereas few studies have already reached or even exceeded one of these parameters [27,85]. However, practically all strength values for CPC in literature were obtained by test methods under static conditions and there are only few reports dealing with the fatigue properties of calcium phosphate cements in load-bearing defect models [108,109]. Hence, testing of cement strength under cyclic loading is one of the most important parameters which needs to be addressed in future research. In addition, since most of the studies on mechanically reinforced biocements were performed with only slowly degradable hydroxyapatite cement matrices and poorly or even non-degradable additives (fibers, polymers), the major challenge for the future is a transfer of the presented concepts to fully degradable materials. This is demanding since degradable cements based on the formation of brushite have harsh setting conditions (low pH, heat release, fast crystallization) and consume a considerable amount of water during setting. Especially, the latter may interfere with the formation of a second hydrogel phase, since the formation of hydrogel and hydrated cement setting product will compete for the available water in the cement liquid.
